# Validation of educational assessments: a primer for simulation and beyond

**DOI:** 10.1186/s41077-016-0033-y

**Published:** 2016-12-07

**Authors:** David A. Cook, Rose Hatala

**Affiliations:** 1 Mayo Clinic Online Learning, Mayo Clinic College of Medicine, Rochester, MN USA; 2 Office of Applied Scholarship and Education Science, Mayo Clinic College of Medicine, Rochester, MN USA; 3 Division of General Internal Medicine, Mayo Clinic College of Medicine, Mayo 17-W, 200 First Street SW, Rochester, MN 55905 USA; 4 Department of Medicine, University of British Columbia, Vancouver, British Columbia Canada

**Keywords:** Lumbar Puncture, Validity Evidence, Validity Argument, Validation Framework, Content Evidence

## Abstract

**Background:**

Simulation plays a vital role in health professions assessment. This review provides a primer on assessment validation for educators and education researchers. We focus on simulation-based assessment of health professionals, but the principles apply broadly to other assessment approaches and topics.

**Key principles:**

Validation refers to the process of collecting validity evidence to evaluate the appropriateness of the interpretations, uses, and decisions based on assessment results. Contemporary frameworks view validity as a hypothesis, and validity evidence is collected to support or refute the validity hypothesis (i.e., that the proposed interpretations and decisions are defensible). In validation, the educator or researcher defines the proposed interpretations and decisions, identifies and prioritizes the most questionable assumptions in making these interpretations and decisions (the “interpretation-use argument”), empirically tests those assumptions using existing or newly-collected evidence, and then summarizes the evidence as a coherent “validity argument.” A framework proposed by Messick identifies potential evidence sources: content, response process, internal structure, relationships with other variables, and consequences. Another framework proposed by Kane identifies key inferences in generating useful interpretations: scoring, generalization, extrapolation, and implications/decision. We propose an eight-step approach to validation that applies to either framework: Define the construct and proposed interpretation, make explicit the intended decision(s), define the interpretation-use argument and prioritize needed validity evidence, identify candidate instruments and/or create/adapt a new instrument, appraise existing evidence and collect new evidence as needed, keep track of practical issues, formulate the validity argument, and make a judgment: does the evidence support the intended use?

**Conclusions:**

Rigorous validation first prioritizes and then empirically evaluates key assumptions in the interpretation and use of assessment scores. Validation science would be improved by more explicit articulation and prioritization of the interpretation-use argument, greater use of formal validation frameworks, and more evidence informing the consequences and implications of assessment.

## Good assessment is important; simulation can help

Educators, administrators, researchers, policymakers, and even the lay public recognize the importance of assessing health professionals. Trending topics such as competency-based education, milestones, and mastery learning hinge on accurate, timely, and meaningful assessment to provide essential information about performance. Assessment of professional competence increasingly extends beyond training into clinical practice, with ongoing debates regarding the requirements for initial and ongoing professional licensure and certification. Front-line educators and education researchers require defensible assessments of health professionals in clinical and nonclinical settings. Indeed, the need for good assessments has never been greater and will most likely continue to grow.

Although workplace-based assessment is essential [1–3], simulation does and will continue to play a vital role in health professions assessment, inasmuch as it permits the targeting of specific topics and skills in a safe environment [4–6]. The conditions of assessment can be standardized across learners, and the spectrum of disease, clinical contexts, and comorbidities can be manipulated to focus on, for example, common yet critical tasks, infrequently seen conditions, activities that might put patients at risk, or situations that provoke specific emotional responses [7, 8]. Thus, it comes as no surprise that simulation-based assessment is increasingly common. A review published in 2013 identified over 400 studies evaluating simulation-based assessments [9], and that number has surely grown. However, that same review identified serious and frequent shortcomings in the evidence supporting these assessments, and in the research studies designed to collect such evidence (i.e., validation studies). The gap between the need for good simulation-based assessment and the deficiencies in the process and product of current validation efforts suggests the need for increased awareness of the current state of the science of validation.

The purpose of this article is to provide a primer on assessment validation for educators and education researchers. We focus on the context of simulation-based assessment of health professionals but believe the principles apply broadly to other assessment approaches and topics.

## Validation is a process

Validation refers to the process of collecting validity evidence to evaluate the appropriateness of the interpretations, uses, and decisions based on assessment results [10]. This definition highlights several important points. First, validation is a process not an endpoint. Labeling an assessment as “validated” means only that the validation process has been applied—i.e., that evidence has been collected. It does not tell us what process was used, the direction or magnitude of the evidence (i.e., was it favorable or unfavorable and to what degree?), what gaps remain, or for what context (learner group, learning objectives, educational setting) the evidence is relevant.

Second, validation involves the collection of validity evidence, as we discuss in a following section.

Third, validation and validity ultimately refer to a specific interpretation or use of assessment data, be these numeric scores or narrative comments [11], and to the decisions grounded in this interpretation. We find it helpful to illustrate this point through analogy with diagnostic tests in clinical medicine [12]. A clinical test is only useful to the degree that (a) the test influences decisions, and (b) these decisions lead to meaningful changes in action or patient outcomes. Hence, physicians are often taught, “Don’t order the test if it won’t change patient management.” For example, the prostate-specific antigen (PSA) test has high reliability and is strongly associated with prostate cancer. However, this test is no longer widely recommended in screening for prostate cancer because it is frequently elevated when no cancer is present, because testing leads to unnecessary prostate biopsies and patient anxiety, and because treating cancers that are found often does not improve clinical outcomes (i.e., treatment is not needed). In other words, the negative/harmful consequences outweigh the beneficial consequences of testing (screening) in many patients [13–15]. However, PSA testing is still useful as a marker of disease once prostate cancer has been diagnosed and treated. Reflecting this example back to educational tests (assessments) and the importance of decisions: (1) if it will not change management the test should not be done, (2) a test that is useful for one objective or setting may be less useful in another context, and (3) the long-term and downstream consequences of testing must be considered in determining the overall usefulness of the test.

## Why is assessment validation important?

Rigorous validation of educational assessments is critically important for at least two reasons. First, those using an assessment must be able to trust the results. Validation does not give a simple yes/no answer regarding trustworthiness (validity); rather, a judgment of trustworthiness or validity depends on the intended application and context and is typically a matter of degree. Validation provides the evidence to make such judgments and a critical appraisal of remaining gaps.

Second, the number of assessment instruments, tools, and activities is essentially infinite, since each new multiple-choice question, scale item, or exam station creates a de facto new instrument. Yet, for a given educator, the relevant tasks and constructs in need of assessment are finite. Each educator thus needs information to sort and sift among the myriad possibilities to identify the assessment solution that best meets his or her immediate needs. Potential solutions include selecting an existing instrument, adapting an existing instrument, combining elements of several instruments, or creating a novel instrument from scratch [16]. Educators need information regarding not only the trustworthiness of scores, but also the logistics and practical issues such as cost, acceptability, and feasibility that arise during test implementation and administration.

In addition, simulation-based assessments are almost by definition used as surrogates for a more “meaningful” clinical or educational outcome [17]. Rarely do we actually want to know how well learners perform in a simulated environment; usually, we want to know how they would perform in real life. A comprehensive approach to validation will include evaluating the degree to which assessment results extrapolate to different settings and outcomes [18, 19].

## What do we mean by validity evidence?

Classical validation frameworks identified at least three different “types” of validity: *content*, *construct*, and *criterion*; see Table 1. However, this perspective has been replaced by more nuanced yet unified and practical views of validity [10, 12, 20]. Contemporary frameworks view validity as a hypothesis, and just as a researcher would collect evidence to support or refute a research hypothesis, validity evidence is collected to support or refute the validity hypothesis (more commonly referred to as the validity argument). Just as one can never prove a hypothesis, validity can never be proven; but evidence can, as it accumulates, support or refute the validity argument.

**Table 1:** The classical validity framework

Type of validity^a^	Definition	Examples of evidence
Content	Test items and format constitute a relevant and representative sample of the domain of tasks	Procedures for item development and sampling
Criterion (includes correlational, concurrent, and predictive validity)	Correlation between actual test scores and the “true” (criterion) score	Correlation with a definitive standard
Construct	Scores vary as expected based on an underlying psychological construct (used when no definitive criterion exists)	Correlation with another measure of the same construct Factor analysis Expert-novice comparisons Change or stability over time

^a^Some authors also include “face validity” as a fourth type of validity in the classical framework. However, face validity refers either to superficial appearances that have little merit in evaluating the defensibility of assessment [26, 59] (like judging the speed of the car by its color) or to influential features that are better labeled content validity (like judging the speed of the car by its model or engine size). We discourage use of the term "face validity"

The first contemporary validity framework was proposed by Messick in 1989 [21] and adopted as a standard for the field in 1999 [22] and again in 2014 [23]. This framework proposes five sources of validity evidence [24–26] that overlap in part with the classical framework (see Table 2). *Content* evidence, which is essentially the same as the old concept of content validity, refers to the steps taken to ensure that assessment items (including scenarios, questions, and response options) reflect the construct they are intended to measure. *Internal structure* evidence evaluates the relationships of individual assessment items with each other and with the overarching construct(s), e.g., reliability, domain or factor structure, and item difficulty. *Relationships with other variables* evidence evaluates the associations, positive or negative and strong or weak, between assessment results and other measures or learner characteristics. This corresponds closely with classical notions of criterion validity and construct validity. *Response process* evidence evaluates how well the documented record (answer, rating, or free-text narrative) reflects the observed performance. Issues that might interfere with the quality of responses include poorly trained raters, low-quality video recordings, and cheating. *Consequences* evidence looks at the impact, beneficial or harmful, of the assessment itself and the decisions and actions that result [27–29]. Educators and researchers must identify the evidence most relevant to their assessment and corresponding decision, then collect and appraise this evidence to formulate a validity argument. Unfortunately, the “five sources of evidence” framework provides incomplete guidance in such prioritization or selection of evidence.

**Table 2:** The five sources of evidence validity framework

Source of evidence	Definition	Examples of evidence
Content	“The relationship between the content of a test and the construct it is intended to measure” [24]	Procedures for item sampling, development, and scoring (e.g., expert panel, previously described instrument, test blueprint, and pilot testing and revision)
Internal structure	Relationship among data items within the assessment and how these relate to the overarching construct	Internal consistency reliability Interrater reliability Factor analysis Test item statistics
Relationships with other variables	“Degree to which these relationships are consistent with the construct underlying the proposed test score interpretations” [24]	Correlation with tests measuring similar constructs Correlation (or lack thereof) with tests measuring different constructs Expert-novice comparisons
Response process	“The fit between the construct and the detailed nature of performance . . . actually engaged in” [24]	Analysis of examinees’ or raters’ thoughts or actions during assessment (e.g., think-aloud protocol) Assessment security (e.g., prevention of cheating) Quality control (e.g., video capture) Rater training
Consequences	“The impact, beneficial or harmful and intended or unintended, of assessment” [27]	Impact on examinee performance (e.g., downstream effects on board scores, graduation rates, clinical performance, patient safety) Other examinee effects (e.g., test preparation, length of training, stress, anxiety) Definition of pass/fail standard

See the following for further details and examples [20, 25, 26]

**Fig. 1:**
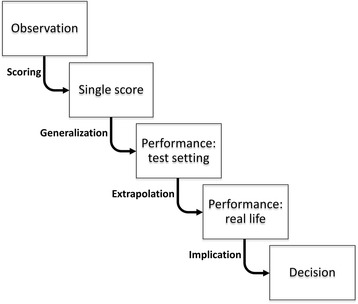
Key inferences in validation

The most recent validity framework, from Kane [10, 12, 30], addresses the issue of prioritization by identifying four key inferences in an assessment activity (Table 3). For those accustomed to the classical or five-evidence-sources framework, Kane’s framework is often challenging at first because the terminology and concepts are entirely new. In fact, when learning this framework, we have found that it helps to not attempt to match concepts with those of earlier frameworks. Rather, we begin de novo by considering conceptually the stages involved in any assessment activity. An assessment starts with a performance of some kind, such as answering a multiple-choice test item, interviewing a real or standardized patient, or performing a procedural task. Based on this observation, a score or written narrative is documented that we assume reflects the level of performance; several scores or narratives are combined to generate an overall score or interpretation that we assume reflects the desired performance in a test setting; the performance in a test setting is assumed to reflect the desired performance in a real-life setting; and that performance is further assumed to constitute a rational basis for making a meaningful decision (see Fig. 1). Each of these assumptions represents an inference that might not actually be justifiable. The documentation of performance (scoring inference) could be inaccurate; the synthesis of individual scores might not accurately reflect performance across the desired test domains (generalization inference); the synthesized score also might not reflect real-life performance (extrapolation inference); and this performance (in a test setting or real life) might not form a proper foundation for the desired decision (implications or decision inference). Kane’s validity framework explicitly evaluates the justifications for each of these four inferences. We refer those wishing to learn more about Kane’s framework to his description [10, 30] and to our recent synopsis of his work [12].

**Table 3:** The validation inferences validity framework

Validity inference	Definition (assumptions)^a^	Examples of evidence
Scoring	The score or written narrative from a given observation adequately captures key aspects of performance	Procedures for creating and empirically evaluating item wording, response options, scoring options Rater selection and training
Generalization	The total score or synthesis of narratives reflects performance across the test domain	Sampling strategy (e.g., test blueprint) and sample size Internal consistency reliability Interrater reliability
Extrapolation	The total score or synthesis in a test setting reflects meaningful performance in a real life setting	Authenticity of context Correlation with tests measuring similar constructs, especially in real-life context Correlation (or lack thereof) with tests measuring different constructs Expert-novice comparisons Factor analysis
Implications/decisions	Measured performance constitutes a rational basis for meaningful decisions and actions	See Table 2, “Consequences”

See Kane [10] and Cook et al [12] for further details and examples

^a^Each of the inferences reflects assumptions about the creation and use of assessment results

Educators and researchers often ask how much validity evidence is needed and how the evidence from a previous validation applies when an instrument is used in a new context. Unfortunately, the answers to these questions depend on several factors including the risk of making a wrong decision (i.e., the “stakes” of the assessment), the intended use, and the magnitude and salience of contextual differences. While all assessments should be important, some assessment decisions have more impact on a learner’s life than others. Assessments with higher impact or higher risk, including those used for research purposes, merit higher standards for the quantity, quality, and breadth of evidence. Strictly speaking, validity evidence applies only to the purpose, context, and learner group in which it was collected; existing evidence might guide our choice of assessment approach but does not support our future interpretations and use. Of course, in practice, we routinely consider existing evidence in constructing a validity argument. Whether old evidence applies to a new situation requires a critical appraisal of how situational differences might influence the relevance of the evidence. For example, some items on a checklist might be relevant across different tasks while others might be task-specific; reliability can vary substantially from one group to another, with typically lower values among more homogeneous learners; and differences in context (inpatient vs outpatient), learner level (junior medical student vs senior resident), and purpose might affect our interpretation of evidence of content, relations with other variables, or consequences. Evidence collected in contexts similar to ours and consistent findings across a variety of contexts will support our choice to include existing evidence in constructing our validity argument.

## What do we mean by validity argument?

In addition to clarifying the four key inferences, Kane has advanced our understanding of “argument” in the validation process by emphasizing two distinct stages of argument: an up-front “interpretation-use argument” or “IUA,” and a final “validity argument.”

As noted above, all interpretations and uses—i.e., decisions—incur a number of assumptions. For example, in interpreting the scores from a virtual reality assessment, we might assume that the simulation task—including the visual representation, the simulator controls, and the task itself—has relevance to tasks of clinical significance; that the scoring algorithm accounts for important elements of that task; that there are enough tasks, and enough variety among tasks, to reliably gauge trainee performance; and that it is beneficial to require trainees to continue practicing until they achieve a target score. These and other assumptions can and must be tested! Many assumptions are implicit, and recognizing and explicitly stating them before collecting or examining the evidence is an essential step. Once we have specified the intended use, we need to (a) identify as many assumptions as possible, (b) prioritize the most worrisome or questionable assumptions, and (c) come up with a plan to collect evidence that will confirm or refute the correctness of each assumption. The resulting prioritized list of assumptions and desired evidence constitute the interpretation-use argument. Specifying the interpretation-use argument is analogous both conceptually and in importance to stating a research hypothesis and articulating the evidence required to empirically test that hypothesis.

Once the evaluation plan has been implemented and evidence has been collected, we synthesize the evidence, contrast these findings with what we anticipated in the original interpretation-use argument, identify strengths and weaknesses, and distill this into a final validity argument. Although the validity argument attempts to persuade others that the interpretations and uses are indeed defensible—or that important gaps remain—potential users should be able to arrive at their own conclusions regarding the sufficiency of the evidence and the accuracy of the bottom-line appraisal. Our work is similar to that of an attorney arguing a case before a jury: we strategically seek, organize, and interpret the evidence and present an honest, complete, and compelling argument, yet it is the “jury” of potential users that ultimately passes judgment on validity for their intended use and context. [31]

It is unlikely that any single study will gather all the validity evidence required to support a specific decision. Rather, different studies will usually address different aspects of the argument, and educators need to consider the totality of the evidence when choosing an assessment instrument for their context and needs.

Of course, it is not enough for researchers to simply collect any evidence. It is not just the quantity of evidence that matters, but also the relevance, quality, and breadth. Collecting abundant evidence of score reliability does not obviate the need for evidence about content, relationships, or consequences. Conversely, if existing evidence is robust and logically applicable to our context, such as a rigorous item development process, then replicating such efforts may not be top priority. Unfortunately, researchers often inadvertently fail to deliberately prioritize the importance of the assumptions or skip the interpretation-use argument altogether, which can result in reporting evidence for assumptions that are easy to test rather than those that are most critical.

## A practical approach to validation

Although the above concepts are essential to understanding the process of validation, it is also important to be able to apply this process in practical ways. Table 4 outlines one possible approach to validation that would work with any of the validity frameworks described above (classical, Messick, or Kane). In this section, we will illustrate this approach using a hypothetical simulation-based example.

**Table 4:** A practical approach to validation

1. Define the construct and proposed interpretation 2. Make explicit the intended decision(s) 3. Define the interpretation-use argument, and prioritize needed validity evidence 4. Identify candidate instruments and/or create/adapt a new instrument 5. Appraise existing evidence and collect new evidence as needed 6. Keep track of practical issues including cost 7. Formulate/synthesize the validity argument in relation to the interpretation-use argument 8. Make a judgment: does the evidence support the intended use?

Imagine that we are teaching first year internal medicine residents lumbar puncture (LP) using a part-task trainer. At the end of the training session, we wish to assess whether the learners are ready to safely attempt an LP with a real patient under supervision.

1. Define the construct and proposed interpretation
  Validation begins by considering the construct of interest. For example, are we interested in the learners’ knowledge of LP indications and risks, their ability to perform LP, or their non-technical skills when attempting an LP? Each of these is a different construct requiring selection of a different assessment tool: we might choose multiple-choice questions (MCQs) to assess knowledge, a series of skill stations using a part-task trainer to asses procedural skill with an Objective Structured Assessment of Technical Skills (OSATS) [32], or a resuscitation scenario using a high-fidelity manikin and a team of providers to assess non-technical skills with the Non-Technical Skills (NOTECHS) scale [33].
  In our example, the construct is “LP skill” and the interpretation is that “learners have fundamental LP skills sufficient to attempt a supervised LP on a real patient.”
2. Make explicit the intended decision(s)
  Without a clear idea of the decisions we anticipate making based on those interpretations, we will be unable to craft a coherent validity argument.
  In our example, our foremost decision is whether the learner has sufficient procedural competence to attempt a supervised LP on a real patient. Other decisions we might alternatively consider include identifying performance points on which to offer feedback to the learner, deciding if the learner can be promoted to the next stage of training, or certifying the learner for licensure.
3. Define the interpretation-use argument, and prioritize needed validity evidence
  In making our interpretations and decisions, we will invoke a number of assumptions, and these must be tested. Identifying and prioritizing key assumptions and anticipating the evidence we hope to find allows us to outline an interpretation-use argument [30].
  In our scenario, we are looking for an assessment instrument in which a “pass” indicates competence to attempt a supervised LP on a real patient. We anticipate that this will involve a physician rating student performance on a skills station. Assumptions in this context include that the station is set up to test techniques essential for LP performance (vs generic skills in sterile technique or instrument handling), that the rater is properly trained, that a different rater would give similar scores, and that learners who score higher on the test will perform more safely on their first patient attempt. Considering the evidence we might need to support or refute these assumptions, and using Kane’s framework as a guide, we propose an interpretation-use argument as follows. We do not know at this stage whether evidence has already been collected or if we will need to collect it ourselves, but we have at least identified what to look for.
  - Scoring: the observation of performance is correctly transformed into a consistent numeric score. Evidence will ideally show that the items within the instrument are relevant to LP performance, that raters understood how to use the instrument, and that video-recording performance yields similar scores as direct observation.
  - Generalization: scores on a single performance align with overall scores in the test setting. Evidence will ideally show that we have adequately sampled performance (sufficient number of simulated LPs, and sufficient variety of conditions such as varying the simulated patient habitus) and that scores are reproducible between performances and between raters (inter-station and inter-rater reliability).
  - Extrapolation: assessment scores relate to real-world performance. Evidence will ideally show that scores from the instrument correlate with other LP performance measures in real practice, such as procedural logs, patient adverse events, or supervisor ratings.
  - Implications: the assessment has important and favorable effects on learners, training programs, or patients, and negative effects are minimal. Evidence will ideally show that students feel more prepared following the assessment, that those requiring remediation feel this time was well spent, and that LP complications in real patients decline in the year following implementation.
  We cannot over-emphasize the importance of these first three steps in validation. Clearly articulating the proposed interpretations, intended decision(s), and assumptions and corresponding evidence collectively set the stage for everything that follows.
4. Identify candidate instruments and/or create/adapt a new instrument
  We should identify a measurement format that aligns conceptually with our target construct and then search for existing instruments that meet or could be adapted to our needs. A rigorous search provides content evidence to support our final assessment. Only if we cannot find an appropriate existing instrument would we develop an instrument de novo.
  We find a description of a checklist for assessing PGY-1’s procedural competence in LP [34]. The checklist appears well suited for our purpose, as we will be using it in a similar educational context; we thus proceed to appraising the evidence without changing the instrument.
5. Appraise existing evidence and collect new evidence as needed
  Although existing evidence does not, strictly speaking apply to our situation, for practical purposes we will rely heavily on existing evidence as we decide whether to use this instrument. Of course, we will want to collect our own evidence as well, but we must base our initial adoption on what is now available.
  We begin our appraisal of the validity argument by searching for existing evidence. The original description [34] offers *scoring* evidence by describing the development of checklist items through formal LP task analysis and expert consensus. It provides *generalization* evidence by showing good inter-rater reliability, and adds limited *extrapolation* evidence by confirming that residents with more experience had higher checklist scores. Other studies using the same or a slightly modified checklist provide further evidence for generalization with good inter-rater reliabilities [35, 36], and contribute extrapolation evidence by showing that scores are higher after training [35, 37] and that the instrument identified important learner errors when used to rate real patient LPs [38]. One study also provided limited *implications* evidence by counting the number of practice attempts required to attain competence in the simulation setting [37]. In light of these existing studies, we will not plan to collect more evidence before our initial adoption of this instrument. However, we will collect our own evidence during implementation, especially if we identify important gaps, i.e., at later stages in the validation process; see below.
6. Keep track of practical issues including cost
  An important yet often poorly appreciated and under-studied aspect of validation concerns the practical issues surrounding development, implementation, and interpretation of scores. An assessment procedure might yield outstanding data, but if it is prohibitively expensive or if logistical or expertise requirements exceed local resources, it may be impossible to implement.
  For the LP instrument, one study [37] tracked the costs of running a simulation-based LP training and assessment session; the authors suggested that costs could be reduced by using trained non-physician raters. As we implement the instrument, and especially if we collect fresh validity evidence, we should likewise monitor costs such as money, human and non-human resources, and other practical issues.
7. Formulate/synthesize the validity argument in relation to the interpretation-use argument
  We now compare the evidence available (the validity argument) against the evidence we identified up-front as necessary to support the desired interpretations and decisions (the interpretation-use argument).
  We find reasonable scoring and generalization evidence, a gap in the extrapolation evidence (direct comparisons between simulation and real-world performance have not been done), and limited implications evidence. As is nearly always the case, the match between the interpretation-use argument and the available evidence is not perfect; some gaps remain, and some of the evidence is not as favorable as we might wish.
8. Make a judgment: does the evidence support the intended use?
  The final step in validation is to judge the sufficiency and suitability of evidence, i.e., whether the validity argument and the associated evidence meet the demands of the proposed interpretation-use argument.
  Based on the evidence summarized above, we judge that the validity argument supports those interpretations and uses reasonably well, and the checklist appears suitable for our purposes. Moreover, the costs seem reasonable for the effort expended, and we have access to an assistant in the simulation laboratory who is keen to be trained as a rater.
  We also plan to help resolve the evidence gaps noted above by conducting a research study as we implement the instrument at our institution. To buttress the extrapolation inference we plan to correlate scores from the simulation assessment with ongoing workplace-based LP assessments. We will also address the implications inference by tracking the effects of additional training for poor performing residents, i.e., the downstream consequences of assessment. Finally, we will measure the inter-rater, inter-case, and internal consistency reliability in our learner population, and will monitor costs and practical issues as noted above.

### Application of the same instrument to a different setting

As a thought exercise, let us consider how the above would unfold if we wanted to use the same instrument for a different purpose and decision, for example as part of a high-stakes exam to certify postgraduate neurologist trainees as they finish residency. As our decision changes, so does our interpretation-use argument; we would now be searching for evidence that a “pass” score on the checklist indicates competence to independently perform LPs on a variety of real patients. We would require different or additional validity evidence, with increased emphasis on generalization (sampling across simulated patients that vary in age, body habitus, and other factors that influence difficulty), extrapolation (looking for stronger correlation between simulation and real-life performance), and implications evidence (e.g., evidence that we were accurately classifying learners as competent or incompetent for independent practice). We would have to conclude that the current body of evidence does not support this argument and would need to either (a) find a new instrument with evidence that meets our demands, (b) create a new instrument and start collecting evidence from scratch, or (c) collect additional validity evidence to fill in the gaps.

This thought exercise highlights two important points. First, the interpretation-use argument might change when the decision changes. Second, an instrument is not “valid” in and of itself; rather, it is the interpretations or decisions that are validated. A final judgment of validity based on the same evidence may differ for different proposed decisions.

## Common mistakes to avoid in validation

In our own validation efforts [39–41] and in reviewing the work of others [9, 25, 42], we have identified several common mistakes that undermine the end-user’s ability to understand and apply the results. We present these as ten mistakes guaranteed to alarm peer reviewers, frustrate readers, and limit the uptake of an instrument.

### Mistake 1. Reinvent the wheel (create a new assessment every time)

Our review [9] found that the vast majority of validity studies focused on a newly created instrument rather than using or adapting an existing instrument. Yet, there is rarely a need to start completely from scratch when initiating learner assessment, as instruments to assess most constructs already exist in some form. Using or building from an existing instrument saves the trouble of developing an instrument de novo, allows us to compare our results with prior work, and permits others to compare their work with ours and include our evidence in the overall evidence base for that instrument, task, or assessment modality. Reviews of evidence for the OSATS [42], Fundamentals of Laparoscopic Surgery (FLS) [43], and other simulation-based assessments [9] all show important gaps in the evidence base. Filling these gaps will require the collaborative effort of multiple investigators all focused on collecting evidence for the scores, inferences, and decisions derived from the same assessment.

### Mistake 2. Fail to use a validation framework

As noted above, validation frameworks add rigor to the selection and collection of evidence and help identify gaps that might otherwise be missed. More important than the framework chosen is the timing (ideally early) and manner (rigorously and completely) in which the framework is applied in the validation effort.

### Mistake 3. Make expert-novice comparisons the crux of the validity argument

Comparing the scores from a less experienced group against those from a more experienced group (e.g., medical students vs senior residents) is a common approach to collecting evidence of relationships with other variables—reported in 73% of studies of simulation-based assessment [9]. Yet this approach provides only weak evidence because the difference in scores may arise from a myriad of factors unrelated to the intended construct [44]. To take an extreme example for illustration, suppose an assessment intended to measure suturing ability actually measured sterile technique and completely ignored suturing. If an investigator trialed this in practice among third-year medical students and attending physicians, he would most likely find a significant difference favoring the attendings and might erroneously conclude that this evidence supports the validity of the proposed interpretation (i.e., suturing skill). Of course, in this hypothetical example, we know that attendings are better than medical students in both suturing and sterile technique. Yet, in real life, we lack the omniscient knowledge of what is actually being assessed; we only know the test scores—and the same scores can be interpreted as reflecting any number of underlying constructs. This problem of “confounding” (multiple possible interpretations) makes it impossible to say that any differences between groups are actually linked to the intended construct. On the other hand, failure to confirm expected differences would constitute powerful evidence of score invalidity.

Cook provided an extended discussion and illustration of this problem, concluding that “It is not wrong to perform such analyses, … provided researchers understand the limitations. … These analyses will be most interesting if they fail to discriminate groups that should be different, or find differences where none should exist. Confirmation of hypothesized differences or similarities adds little to the validity argument.” [44]

### Mistake 4. Focus on the easily accessible validity evidence rather than the most important

Validation researchers often focus on data they have readily available or can easily collect. While this approach is understandable, it often results in abundant validity evidence being reported for one source while large evidence gaps remain for other sources that might be equally or more important. Examples include emphasizing content evidence while neglecting internal structure, reporting inter-item reliability when inter-rater reliability is more important, or reporting expert-novice comparisons rather than correlations with an independent measure to support relationships with other variables. In our review, we found that 306/417 (73%) of studies reported expert-novice comparisons, and 138 of these (45%) reported no additional evidence. By contrast, only 128 (31%) reported relationships with a separate measure, 142 (34%) reported content evidence, and 163 (39%) reported score reliability. While we do not know all the reasons for these reporting patterns, we suspect they are due at least in part to the ease with which some elements (e.g., expert-novice comparison data) can be obtained.

This underscores the importance of clearly and completely stating the interpretation-use argument, identifying existing evidence and gaps, and tailoring the collection of evidence to address the most important gaps.

### Mistake 5. Focus on the instrument rather than score interpretations and uses

As noted above, validity is a property of scores, interpretations, and uses, not of instruments. The same instrument can be applied to different uses (the PSA may not be useful as a clinical screening tool, but continues to have value for monitoring prostate cancer recurrence), and much validity evidence is context-dependent. For example, score reliability can change substantially across different populations [44], an assessment designed for one learning context such as ambulatory practice may or may not be relevant in another context such as hospital or acute care medicine, and some instruments such as the OSATS global rating scale lend themselves readily to application to a new task while others such as the OSATS checklist do not [42]. Of course, evidence collected in one context, such as medical school, often has at least partial relevance to another context, such as residency training; but determinations of when and to what degree evidence transfers to a new setting are a matter of judgment, and these judgments are potentially fallible.

The interpretation-use argument cannot, strictly speaking, be appropriately made without articulating the context of intended application. Since the researcher’s context and the end-user’s context almost always differ, the interpretation-use argument necessarily differs as well. Researchers can facilitate subsequent uptake of their work by clearly specifying the context of data collection—for example, the learner group, task, and intended use/decision—and also by proposing the scope to which they believe their findings might plausibly apply.

It is acceptable to talk about the validity of scores, but for reasons articulated above, it is better to specify the intended interpretation and use of those scores, i.e., the intended decision. We strongly encourage both researchers and end-users (educators) to articulate the interpretations and uses at every stage of validation.

### Mistake 6. Fail to synthesize or critique the validity evidence

We have often observed researchers merely report the evidence without any attempt at synthesis and appraisal. Both educators and future investigators greatly benefit when researchers interpret their findings in light of the proposed interpretation-use argument, integrate it with prior work to create a current and comprehensive validity argument, and identify shortcomings and persistent gaps or inconsistencies. Educators and other end-users must become familiar with the evidence as well, to confirm the claims of researchers and to formulate their own judgments of validity for their specific context.

### Mistake 7. Ignore best practices for assessment development

Volumes have been written on the development, refinement, and implementation of assessment tasks, instruments, and procedures [23, 45–48]. Developing or modifying an assessment without considering these best practices would be imprudent. We could not begin to summarize these, but we highlight two recommendations of particular salience to health professions educators, both of which relate to content evidence (per the classic or five sources frameworks) and the generalization inference (per Kane).

First, the sample of tasks or topics should represent the desired performance domain. A recurrent finding in health professions assessment is that there are few, if any, generalizable skills; performance on one task does not predict performance on another task [49, 50]. Thus, the assessment must provide a sufficiently numerous and broad sample of scenarios, cases, tasks, stations, etc.

Second, the assessment response format should balance objectification and judgment or subjectivity [51]. The advantages and disadvantages of checklists and global ratings have long been debated, and it turns out that both have strengths and weaknesses [52]. Checklists outline specific criteria for desired behaviors and guidance for formative feedback, and as such can often be used by raters less familiar with the assessment task. However, the “objectivity” of checklists is largely an illusion; [53] correct interpretation of an observed behavior may yet require task-relevant expertise, and forcing raters to dichotomize ratings may result in a loss of information. Moreover, a new checklist must be created for each specific task, and the items often reward thoroughness at the expense of actions that might more accurately reflect clinical competence. By contrast, global ratings require greater expertise to use but can measure more subtle nuances of performance and reflect multiple complementary perspectives. Global ratings can also be designed for use across multiple tasks, as is the case for the OSATS. In a recent systematic review, we found slightly higher inter-rater reliability for checklists than for global ratings when averaged across studies, while global ratings had higher average inter-item and inter-station reliability [52]. Qualitative assessment offers another option for assessing some learner attributes [11, 54, 55].

### Mistake 8. Omit details about the instrument

It is frustrating to identify an assessment with relevance to local needs and validity evidence supporting intended uses, only to find that the assessment is not specified with sufficient detail to permit application. Important omissions include the precise wording of instrument items, the scoring rubric, instructions provided to either learners or raters, and a description of station arrangements (e.g., materials required in a procedural task, participant training in a standardized patient encounter) and the sequence of events. Most researchers want others to use their creations and cite their publications; this is far more likely to occur if needed details are reported. Online appendices provide an alternative to print publication if article length is a problem.

### Mistake 9. Let the availability of the simulator/assessment instrument drive the assessment

Too often as educators, we allow the availability of an assessment tool to drive the assessment process, such as taking an off-the-shelf MCQ exam for an end-of-clerkship assessment when a performance-based assessment might better align with clerkship objectives. This issue is further complicated with simulation-based assessments, where the availability of a simulator may drive the educational program as opposed to designing the educational program and then choosing the best simulation to fit the educational needs [56]. We should align the construct we are teaching with the simulator and assessment tool that best assess that construct.

### Mistake 10. Label an instrument as validated

There are three problems with labeling an instrument as validated. First, validity is a property of scores, interpretations, and decisions, not instruments. Second, validity is a matter of degree—not a yes or no decision. Third, validation is a process, not an endpoint. The word validated means only that a process has been applied; it does not provide any details about that process nor indicate the magnitude or direction (supportive or opposing) of the empiric findings.

## The future of simulation-based assessment

Although we do not pretend to know the future of simulation-based assessment, we conclude with six aspirational developments we hope come to pass.

1. We hope to see greater use of simulation-based assessment as part of a suite of learner assessments. Simulation-based assessment should not be a goal in and of itself, but we anticipate more frequent assessment in general and believe that simulation will play a vital role. The choice of modality should first consider what is the best assessment approach in a given situation, i.e., learning objective, learner level, or educational context. Simulation in its various forms will often be the answer, especially in skill assessments requiring standardization of conditions and content.
2. We hope that simulation-based assessment will focus more clearly on educational needs and less on technology. Expensive manikins and virtual reality task trainers may play a role, but pigs feet, Penrose drains, wooden pegs, and cardboard manikins may actually offer more practical utility because they can be used with greater frequency and with fewer constraints. For example, such low-cost models can be used at home or on the wards rather than in a dedicated simulation center. As we consider the need for high-value, cost-conscious education [57], we encourage innovative educators to actively seek low-tech solutions.
3. Building off the first two points, we hope to see less expensive, less sophisticated, less intrusive, lower-stakes assessments take place more often in a greater variety of contexts, both simulated and in the workplace. As Schuwirth and van der Vleuten have proposed [58], this model would—over time—paint a more complete picture of the learner than any single assessment, no matter how well-designed, could likely achieve.
4. We hope to see fewer new assessment instruments created and more evidence collected to support and adapt existing instruments. While we appreciate the forces that might incentivize the creation of novel instruments, we believe that the field will advance farther and faster if researchers pool their efforts to extend the validity evidence for a smaller subset of promising instruments, evaluating such instruments in different contexts, and successively filling in evidence gaps.
5. We hope to see more evidence informing the consequences and implications of assessment. This is probably the most important evidence source, yet it is among the least often studied. Suggestions for the study of the consequences of assessment have recently been published [27].
6. Finally, we hope to see more frequent and more explicit use of the interpretation-use argument. As noted above, this initial step is difficult but vitally important to meaningful validation.
